# Mobile care - a possible future for emergency care in Sweden

**DOI:** 10.1186/s12873-023-00847-1

**Published:** 2023-07-27

**Authors:** Christofer Teske, Ghassan Mourad, Micha Milovanovic

**Affiliations:** 1 Department of Health, Medicine and Care (HMV), Faculty of Medicine and Health Sciences, Linköping University, Linköping, Norrköping, SE-601 74 Sweden; 2 Department of Emergency, Vrinnevi Hospital, Norrköping, Sweden; 3 Department of Internal Medicine, Vrinnevi Hospital, Norrköping, Sweden

**Keywords:** Mobile care, Mobile team, Emergency care, Patient safety

## Abstract

**Introduction:**

Provision of mobile care at the home of patients appears to become necessary as the population becomes increasingly older. But there are challenges in moving emergency care from hospitals to the home of patients. The aim of the study was therefore to describe the experiences of the mobile care in Sweden.

**Method:**

Semi structured interviews were conducted with 12 persons with experience of mobile care in Sweden, such as nurses, physicians, civil servants and politicians. Qualitative latent content analysis was used as an analysis method.

**Result:**

The results show that cooperation is of utmost importance to achieve functioning mobile care. Cooperation both on an inter-organizational level and on a close team-work is required for all of the involved parties in mobile care to take on a joint responsibility for the patient. As mobile care is primarily provided to elderly multimorbid patients, a comprehensive view on patient care is required in which the patient and their relatives experience security.

**Conclusion:**

Mobile care is seen as a moving care that comes to the seeking person and not the other way around. The resources are distributed where they make the most use, that is, closest to the individual. Mobile care is seen as a complement to the traditional hospital care. This means a different way of working that requires close collaboration between different categories of personnel and organizations, where there should not be any discussions about boundaries, rather, the discussion should include patient’s needs and situation instead.

## Introduction

Due to progress in medical research and economic conditions, people are getting older today. An increase in age also contributes to an increase in the number of diseases and number of medications, all of which leading to older persons getting a complex disease picture [1]. The care of elderly patients in Swedish emergency departments has proved to be deficient, which is partly since the patient’s reason for visiting is often not life-threatening and is thus not a high priority. Long waiting times entail a risk of care-related injuries, which is also of concern [2]. Older people are more prone to falls, depression, and cognitive impairment, and often intake of multiple medications. These circumstances complicate the investigation and management of older persons in the emergency department [3]. Nursing care is prioritized away in favour of patients with urgent care needs. It creates frustration in the nurse, and the feeling of inadequacy and loss of control occurs at the same time as the risk of medical injuries increases [4, 5]. The pressure on a reduced number of care places in hospitals, increased population growth, and an aging population mean that caring in the home is an increasingly necessary service.

Person-centered care is an approach that puts the person receiving care at the center. This means that the patient’s own participation is important, and that care is based on the patient’s unique needs and abilities. The staff around the patient needs to work together to achieve common goals, and person-centered care ensures that the patient is treated with respect and dignity. To work with person-centered care, good structures, and competent staff who can actively implement knowledge about person-centered care in practice are also needed [6]. Therefore, providing care in the home of patients, such as mobile care, could be a possible way to enhance greater participation from patients. In this way, healthcare resources can also be used more efficiently. The goal is a person-centred care that strengthens health and prevents physical and mental illness.

### Bringing the hospital to the patient: the mobile care

Mobile care as a term refers to care that is provided on site at the patient and thus constitutes an alternative to the emergency department and or hospital admission. This type of care is considered as a safe and effective option to hospital care [7] and compared to traditional hospital care, patients show higher satisfaction with mobile care [1, 8]. There are different names for “Mobile care” in the world, but despite this the principle is similar. For instance, in Australia, “Hospital in the Home” is used as an opportunity for patients to receive emergency care at home instead of in hospital. It is an opportunity for patients to completely avoid hospital care or to be able to be discharged from hospital earlier and be connected to the Mobile care-unit. Diagnoses that are treated via the mobile device include infections and venous thrombosis. A model in England that is based on the fact that interventions that usually require hospital care are now being moved to the home. By moving the care to the patient and thus avoiding transferring the patient between hospitals has shown increased clinical effect and is more cost-effective [1, 9].

### The mobile care that is conducted in Sweden today

Alternatives to hospital care are discussed at the government level in Sweden [10]. There is an agreement to further develop a “close care” in 2022. The goal of this change is that the healthcare system should contribute to the patient receiving good, close, and coordinated care that strengthens health. The goal is also for the patient to participate based on their conditions and preferences and to create a more economically efficient and long-term sustainable healthcare system. However, in Sweden, there is no structured model for how to provide close care. Examples of close care are mobile care, i.e., there are some different mobile care teams that all aim to avoid hospital admissions for elderly patients with multi-morbidity and who need acute care [11–13]. These teams are normally connected to either emergency or geriatric clinics.

It is a challenge to move the care of acute ill patients from the emergency rooms to the patient’s home related to patient safety and the limitations that exist in the patient’s home. If mobile care is to be a part of emergency care nationally and internationally, more research is required in this area. An increased knowledge of how mobile care is experienced can provide an improved and changed understanding of how this should be implemented to be part of the future health care. The aim of the current study was to describe experiences of mobile care in Sweden.

## Method

### Participants and recruitment

The study was approved by the Southeast Ethics Committee. Linköping, Sweden (reg. number: 548–2019) and informed consent was obtained from all participants. Inclusion criteria were persons who worked with or did have a great knowledge/experience regarding mobile care teams in Sweden. In order to get relevant informants, 15 health care strategists in south-eastern Sweden were contacted, 3 of these did not want to participate, which resulted in 12 informants who were interviewed. The distribution was as follows; four persons were healthcare workers, five were politicians or civil servants/women with positions of trust and the remaining three were heads of units. Of the participants, 6 were women and 6 were men.

### Data collection

Data collection was carried out in the spring of 2019. Due to the geographical spread of participants, eight of the participants were interviewed via Microsoft® Skype® (California, U.S.A.). The interviews that were conducted physically took place at the informants workplace, a place that the participants themselves had chosen. An open-ended interview guide, Table 1, consistent with the study aim was used in the study. The interview guide was revised during the study in order to be able to answer the aim. All interviews lasted between 30 and 65 min and were audio-taped and later transcribed verbatim. These interviews were recorded with an Olympus-WS-853 voice recorder (Tokyo, Japan). For the interviews conducted via Microsoft® Skype® (California, U.S.A.), the same program was used for recording.

**Table 1:** Shows the interview guide that was used in the study

Topic	Questions
Clinic/Organization	• Can you describe what mobile care means to you? • What is the name of the mobile care in which you operate? • Do you want to describe the department? What occupational categories are included in mobile care? Is the unit part of a fixed-term project or a long-term organization?
Target group:	• Can all people take part in mobile care? What inclusion criteria are required to be cared for by mobile care? Can you describe which patients you care for in mobile care? • How do patients get in touch with the mobile device if they are in need of (emergency) care? • How many patients visits / year? How many patients are included in the mobile care (if it is a limited quantity).
Working environment:	• Can you describe a normal working day. What is included in the main tasks of mobile care? •The mobile care uses decision support for choosing the optimal level of care for the emergency sick, injured patients (If so which, can you tell me more about it.) • How do you experience patient safety? Can you describe in what way?
Cooperation:	• Is there any collaboration with other units or organizations? And in that case how does this collaboration take place?
Potential for improvement:	• How do you see the present and the future of mobile care? Benefits and disadvantages. • Do you have something more you want to add? Based on what the informant says, the authors then ask follow-up questions to understand more. To the people who do not work actively in mobile care, we will ask them to describe mobile care from their perspective and focus on follow-up questions. Do you agree to be contacted for a follow-up interview about the mobile the care unit? (Yes / No, if yes write down contact information)

### Data analysis

For the analysis, a qualitative latent content analysis with an inductive approach was used in the current study [14]. The transcribed content was read through several times by the authors to create entirety of the material. In the text, meaningful units were identified that corresponded to the aim of the study, thereafter, the meaningful unit was condensed into shorter sentences. By creating an abstract image of the short sentences, a code was set by the authors who thus described the content. Codes that were similar formed subcategories and main categories. The content of the categories is closely related but differs from the content of the other categories. A theme then emerged based on the subcategories and main categories that had previously been identified [14]. The theme that was formed then formed the basis for headings in the results of the study. Table 2 provides examples of the various phases of the analysis. Quotations are used to strengthen the results. These are drawn from the raw data and translated into English.

**Table 2:** Shows examples of the different phases of the analysis

Meaningful unit	Condensed sentence unit	Code	Subcategory	Main category
That we can offer better care and we can avoid a lot of care injuries. Many people get medical injuries because they are hospitalized. Most people feel better about being at home. Especially if you are multi-sick. They do not feel so well in hospital. If they can get good care at home, it is worth a lot.	The advantage is that we can offer better care and avoid healthcare injuries.	Avoid healthcare injuries	Patient safety	Holistic view of the patient
Then it is better to provide the services as close to the patient as possible and then often at home. Then you get to know how this person works in his entire life situation.	Provide services close by and often at home so that the medical staff are able to get an entire picture and understanding of the persons entire life situation	The patient in a home environment	Overall picture	Holistic view of the patient

## Results

Based on the analyzed data, an overall theme emerged that is presented together with the three main categories and associated sub-categories in Table 3. The interviews were referred to in the results using the numbers 1–12 to preserve their confidentiality.

**Table 3:** Shows the analysis process carried out according to Patton (2015), [14]

Theme	A holistic care given through collaboration in the patient’s home environment		
**Main categories**	Boundless collaboration	Resources in the local environment	Holistic View of the Patient.
**Subcategories**	Collaboration across organizational boundaries	Measures and treatments at home	Create security
	Team collaboration	Function description - mobile teams	Overall picture
	Shared responsibility	Target group	Patient safety
	Competence	Resources	
		Healthcare is moving home	

### Boundless collaboration

This main category contains subcategories that describe how the organization works and how teamwork makes it possible to work across organizational boundaries for the patient’s best interests.

### Collaboration across organizational boundaries

Mobile care that is conducted today is a common initiative between municipalities, regions, and primary care to improve care for the patient. Increased resources at the health centres have been distributed to be able to deploy mobile home care physicians, as well as physicians and nurses from the specialist health care at the hospitals. The informants described the following on the subject;

*“There are many actors, cultures and organizations that will collaborate and be out with the patients outside the hospital walls. It is a great challenge” (1).*

Should the patient need hospital care, the patient can be admitted directly from the home to a nursing department at a hospital, instead of having to manage the long waiting times of the emergency department. Direct admission is possible both from the mobile team and from home health care. Then direct contact is made with the emergency department at the hospital. The following is described;

*“It is also possible to admit the patient directly to the hospital, if necessary, in order to avoid the patient ending up in the emergency department” (5).*

It was equally important that the management structure is good and that the employees have the trust and permission to enter each other’s functions, even at managerial level. This is to avoid demarcations and agreements but through cooperation to obtain synergy effects that are positive for the patient. The following parts were highlighted as examples;

*“It’s about how we convey our services, it’s a different way of working outside the hospital? walls, if you work in the patient’s home you have to cooperate in a completely different way than you do in traditional care, it is a great cultural work, where we do not talk about boundaries, but based on the patient’s needs and situation” (3).*

### Team collaboration

The collaboration between different organizations and units was perceived to provide a good exchange of knowledge between the health care staff and different professional categories. By having separate physicians who work with the home health care patients, this avoids them getting involved in the regular care center operations. This gives the physicians more time for direct work with municipal nurses, assistant nurses or more time at the patient’s home. The communication paths have therefore proved to be simpler and save a lot of time. In the past, nurses often found it more difficult to obtain contact with physicians, so that work with patients could progress, but now physicians and nurses work in the same team and have common visits or continuous contacts. Two of the informants talks about this;

*“It was seen that the municipal nurses had poor or non-existent contact with the health centre’s doctors. The municipal nurses never received help from the doctor in question for a medical assessment or help with medication prescriptions” (7).*

### Shared responsibility

The patients who are involved in mobile care usually have the home care service or home health care as a basis, so it is usually the municipal nurse in home health care who, together with the home health care physician, has the overall patient responsibility. If the time is not sufficient for the municipal nurse, the nurse in the mobile team should be able to help with the other patients that the units have in common. The results from the interviews showed that there are difficulties in making mobile care work. Above all, there are care cultures barriers that must be reconsidered. For example, the healthcare staff is in the habit of acting only within their specific area of responsibility. Therefore, it is important to be able to give and take in situations that involve doing someone else’s work or taking over an area that belongs to another care unit. It is a shared responsibility in order to make it as good as possible for the patient.

### Competence

The right skills are required for a functioning mobile care. The professional health care staff are usually physicians and nurses involved in the mobile team. The individual should be comfortable working in a less controlled environment and be flexible in order to be suitable to work within the mobile teams. The mobile team’s physicians are often specialists in geriatrics or internal medicine. However, it is more important that the physician in the team is experienced and interested, than being a specialist in a certain field. Several of the physicians rotate between the mobile team and the usual work at the hospital. It has generated in an increased knowledge that the physicians benefit from in the hospital because they are then aware of what the patient is being discharged to. The interviews showed that the physician thus gets an increased holistic view of the patient and can carry out better planning before discharge from the hospital. The nurses in the mobile teams should be specialist trained or have long work experience. In the interviews, it appears that there is additional competence available, in the form of a backup physicians, if the situation requires.

*“I mean we have primary care physicians who are happy to undertake and are responsible for fairly advanced medical measures and others feel very uncomfortable with all infusions and injections”* (3).

### Resources in the local environment

The second main category contains subcategories such as business descriptions and the treatment options that the mobile teams have.

### Measures and treatments at home

There should be a careful consideration of whether a patient needs highly specialized medical care or whether he can be treated at home. The primary assessment can be done at home with anamnesis, vital parameters, and possible sampling where test results for blood gas can be obtained directly in connection to the patient. Much of what is done today in hospitals could be handled by a mobile care unit. One of the informants describes this as follows;

*“If you are going to be in hospital, it is because you need some form of urgent action, for example that your heart stops. Then it is not so good if you are at home. Then it is better to be in hospital to be continuously monitored. Otherwise, if you want to monitor someone’s heart rhythm without causing any action, then you can just as easily do it at home”* (10).

Common diagnoses treated in mobile care are infections, heart failure and electrolyte disturbances. In terms of treatment, most things are possible at home. Examples of treatments are intravenous diuretics, antibiotics, pain relief or blood transfusions. It is important that healthcare professionals who do not usually work in mobile healthcare know what treatment options are available in order to be able to offer it to patients who may be relevant for treatment at home. As another informants describes this;

*“I have supervised many physicians. What are the treatment options, etc. at home so they get an idea of what you can do at home” (7).*

### Function description - mobile teams

In current study, most of the mobile teams usually start from a hospital and work office hours. On evenings and weekends, the home care team takes over. To create an idea of the patients who belong to the teams in mobile care, the patient’s condition is discussed, just like in hospitals but at home. It is physicians and nurses from the hospital who participate and if possible, the municipal nurse must attend. There must be a clear care plan for the patient and there is proactive planning so that all health care staff can feel safe in their work.

Health care staff visits the patient on planned visits but can at any time be called by a patient or a health care staff who believes that the patient needs an emergency visit. The mobile teams often want a primary assessment to be performed by the municipal nurse, who then contacts the mobile team. The following description is provided;

*“8–17, Monday to Friday, they can always get in touch with us via their municipal nurse who must first be contacted to make an initial assessment. The goal is for there to be a clear plan, so the municipal nurse knows what to do if something happens” (5).*

### Target group

All patient categories may belong to mobile care in various forms. It is the need that governs who can be assessed to the mobile care. It can be about demographic or geographical circumstances that make a certain target group suitable or not. As illustrated by one informant;

*“It is probably the need that governs and not what is in the passport or in the medical history. It can be an asthmatic who is twenty years old or a COPD patient who is 90 years old” (11).*

To be included in mobile care, it should not be the diagnosis or age, but the need and the situation. To increase public health, healthcare should focus on including vulnerable groups. This means that care must find flexible solutions to reach a target group that otherwise does not seek care in the form of care that we are normally used to, a health center or a hospital. As illustrated by another informant;

*“The population today is heterogeneous and then you have to have flexible solutions to integrate vulnerable and marginalized groups and their mobile care can have a special place in care” (1).*

### Resources

To conduct mobile care, it should be efficient, i.e., it must be resource efficient to the extent that it uses healthcare staff in an efficient manner and that it must be cost-effective. Mobile care is effective in the sense that inpatient hospital care can be avoided, as well as visits to the emergency department. It is not resource efficient because it cares for few patients per unit of time. The following descriptions are provided by informants;

*“We really see that it has good effects. Then it is important to see that it is economically efficient and that we can use the resources we have and that it will not just be independent projects. Without being able to implement it in a good way” (6).*

The need is perceived as greater for the mobile teams than there are currently resources for. There is a great need for care for those who do not always can visit the hospital.

### Healthcare is moving home

Mobile care units are units that are coming to the patient instead of the patient seeking care. The initiatives from these specialties are based on the knowledge that people spend most of their lives at home and not in hospitals. Because that is when you see how the patient functions in their entire life situation. This statement is illustrated by one informant;

*“I think you must understand that just because care is mobile does not mean that it is the same level of care. It does not have to be the same level of primary care, but it can be highly specialized care. I think that people understand this better if you exemplify with, for example, hospital at home, regular hospital activities that can also be performed outside the physical hospital building” (1).*

Mobile care should be a care alternative, a complement to traditional care. This means that the care moves the resources where they do the most good, i.e., closest to the patient. The lack of inpatient care places contributes to the need to construct alternative forms of care. This is reproduced with the following two quotes;

*“I do not think we will have more hospital beds in the future, but we will need to care for more and more patient groups at home” (3)*.

### Holistic view of the patient

The third category is called the Holistic View of the Patient. The category highlights the need for care to be based on person-centered care and sees the whole person and their needs.

### Create security

In order to create security for a patient who is cared for at home, one of the prerequisites is that the care must be accessible. Patients must have the opportunity to contact or be able to talk to someone about their health condition. It is also the staff who care for the patient at home who should feel secure. For health care staff to feel safe with the situation surrounding the patient, there must be a clear plan and the possibility to contact a physician in case of deterioration.

For relatives to be able to create security for the patient, information to the patient and relatives was important. By not only seeing the patient’s illness but the whole patient, which includes the patient’s social network, relatives gained knowledge of the patient’s condition and thus contributed to increased trust in the care that was provided. Participants in the study believe that people feel safe when they are cared for in their homes. To create security, human presence was a contributing factor. Experiencing security, however, can be different for people. As illustrated by one informant;

*“Yes, I would like to say that the patient feels better and is safer in their home if they have people around them…, The security can be that you experience it when you always have people around you. Or that it may consist of someone coming and looking at you” (7).*

The hospital is a place where you can find staff around the clock, but that does not mean that human presence is a fact for the patient. The lack of health care staff was not considered something negative for safety because it does not have to be care staff who are close to the patient. It could be relatives or home care staff. One informant describes this as follows;

*“But you must always deliver security, security is a component in all healthcare. And to deliver security, you do not have to be a healthcare professional. It can be the case that you are in a human context, so to speak. If you need to be looked at or you are worried or just need someone to call or someone to ask for advice” (2).*

### Overall picture

When the patient is in their home environment, it is perceived as easier for the healthcare staff to perform assessments compared to whether patients were cared for in a hospital setting. It is easier to convey sensitive information when a patient is at home. The patient’s home environment seems to facilitate an assessment by the healthcare staff at the same time as the patient is in a comfortable environment to share information about their situation. One informant describes this as follows;

*“When you have a patient at home, you can make much safer assessments as well, because if the patient comes to a room in the hospital, that’s not how it works for the patient here at home, is it?“ (3).*

The interviews highlighted how important it is to start from a person-centered care where human needs are in focus rather than putting the patients into different compartments. Another study participant describes this as follows;

*“The important thing in this is person-cantered care, ie the care should be based on what this patient needs, it may not be fine-tuned diabetes but it may be pain relief and anxiety relief that is the most important, so you focus on what is important in conversations with patients” (8).*

The results from the interviews also showed that healthcare easily places people in different compartments, but this does not reflect the patient’s reality. And yet another study participant;

*“We must move away from dividing care into special organs, diagnoses, buildings and instead, what works for people in their lives” (1).*

### Patient safety

Patients who were part of the mobile teams often had multiple illnesses and required care from many different parts of the health care system. It turned out that patients do not always have someone who can help contact the health care when needed or not able to book a suitable transport in case of illness or a feeling of wanting to be assessed by a healthcare professional. This can mean that the patient waits too long to seek care and results in unnecessary deterioration of their health. This can then be prevented by a mobile team assessing the patient in their home and have an overall view. The following description are provided by one informant;

*“It will be a unreported cases there that ends up between the chairs because there is really no one to see so they come to a health centre and it is too cumbersome to book a lying transport or a medical trip… Which means that many do not seek help until they are so bad as they have to get to the emergency room” (9).*

The results show several examples of healthcare injuries that can be prevented in a hospital environment. Patient safety was stated as high and in favour of care in the home environment. A statement from one informant;

*“You probably reduce the risk of healthcare-associated infections. Just if we think in inpatient care with reduced disruption, anxiety, etc. with single rooms, it is even better at home, etc. You probably reduce the consumption of sleeping pills and reduce the incidence of falls. They are in an above environment. So I think more potential in it than threats” (10).*

It should always be possible for the patient to go to the hospital if the situation requires it. It was seen as a prerequisite if patient safety is to be experienced as high by the staff who work with patients at home. Since there are limited opportunities for monitoring the patient’s vital parameters in the home, it is seen as negative from a safety perspective. The participants agree that through digital solutions, there are conditions to monitor the patient at home. A further statement from a informant;

*“The risks are that you make a point effort. And has no possibility of monitoring. At least in the current situation. It certainly comes with digitization” (11).*

## Discussion

The results of the study show that mobile care is seen as a mobile care that comes to the care seeker and not the other way around. The resources are distributed where they do the most good, i.e., closest to the individual. Mobile care is also seen as a complement to traditional hospital care. This means a different way of working that requires close cooperation between different staff categories and organizations, where it is not discussed about boundaries but about the patient’s needs and situation. The interviewees say that mobile care is a way of care where one has to cooperate across borders and help each other to get a better holistic view of the care of the patient.

A prerequisite for mobile care to work is that collaboration across organizational boundaries must be improved and should thus be something to strive for in the future. To be able to provide good care, it is necessary for the care staff to look beyond the organizational boundaries and instead focus on the patient. Mobile care should not be seen as a new competing organization but as an alternative form of care for already existing parts of the health care system. Brody et al. [15] have investigated which barriers are considered when alternatives to in patient emergency care are designed and are to be implemented. In their case, it is about “Hospital at home”, where emergency care is provided in the patient’s home. They believe that good cooperation is required between the various actors in such an organization. It is both about managers for the actors sitting and discussing the work with the patient, but also that the cooperation between the care staff closest to the patient works in a good way. This is confirmed by Kuriakose et al. [16] who believe that the collaboration between hospitals, primary care and specialist care should be improved and can result in increased patient safety. This suggests that with a well-thought-out structure and good cooperation with the various actors in healthcare, there are good conditions for mobile healthcare units to be established in the health care system.

Mobile care must not only be efficient for the patient but also be both resource efficient in so far as it uses healthcare staff and equipment in an efficient manner and that it must be cost-effective. According to Edmond et al. [17] it is proven to be more cost-effective to care for the patient at home than in the hospital. Thus, home care proved to be a good strategy both for the patient’s health and in order to work more cost-effectively. The author believes that it is important to innovate and to look at both the cost perspective and the patient perspective, but also the work environment for the staff when managing mobile care units. A visit by a mobile team at home should reasonably cost less than a day of care in a hospital. More research in this topic that supports the implementation of mobile care could contribute to this being applied to a higher degree. The hope is that mobile care would be a more cost-effective solution compared to traditional hospital care. In this way, healthcare resources can also be used more efficiently. Then there would be very large gains to be made at the individual, organizational and societal level. A greater participation from patients would also be possible.

The results of the study showed that the experience of mobile care is that it provides good conditions for the care to be conducted with a holistic view of the patient. It is an advantage to see the patient in their home environment because the person is placed in the context in which they normally live. Tiberg et al. [18] concluded that it is beneficial to see the patient in their home environment as the care staff could handle risk factors and deal with problems that arose in the patient’s everyday life. Li et al. [19] and Lee et al. [1] reported that patients felt safer in their own homes than in hospitals. At the same time, some of the patients felt that hospital care was safer because they have better equipment and a higher level of competence. Furthermore, they indicated that the availability of care and human presence increased the security of the patients who are cared for at home. This is confirmed by Molina-Mula et al. [20] just as the authors, believe that human presence is related to the patient’s sense of security. Whether patients are at home or in a hospital, the presence of healthcare staff is not always guaranteed. If it is the degree of available human presence that is decisive for the patient to feel safe is interpreted as an individual.

The caring relationship is characterized by a professional commitment on the part of the caregiver, which means that the focus is on the patient’s need for care [6]. The results of this study showed that mobile care is a care alternative where patients feel secure and that it is important to start from each individual and to see their needs. Breiholtz et al. [21] describe that older people find it difficult to influence decisions that have already been made at an organizationally high level regarding their own care and are then prevented from having their wishes and needs fulfilled. The authors’ interpretation is that mobile care constitutes a safe care alternative if the care is provided in connection with the patient. It is also possible that mobile care can improve patients’ quality of care, efficiency, and health outcomes.

### Method discussion

A strength of this study is that the authors used strategically selected participants from different professional categories from similar units and with different geographical residences based on their knowledge or experience in the field. Different professional categories were interviewed to get a spread in the sample and to capture different views on how the mobile care is structured and carried out. The different geographical areas were selected to get a wider picture of current phenomena.

Another strength of the current study was the interview guide, it was designed to be neither leading nor evaluative. Each interview ended by asking if the informants felt that there was any additional thought that arose from the question area and that was not addressed. This was used to open up for them to speak freely and add additional thoughts that have not previously appeared in the requested area. The informants were also informed that they were happy to get in touch if, after the end of the interview, they came up with anything else that they thought was important regarding the topic. The fact that the informants repeatedly received information, both verbally and in writing, about the purpose of the study and that the material cannot be traced back to them personally may have had a positive effect on them feeling more comfortable in the interview situation. Overall, this made it possible for the study to achieve confidence in the credibility, and convincing results from the participants’ perspective.

The condensation of data into meaningful units was performed individually by the authors to then compare each other’s results. To limit the influence of the authors’ perceptions in the analysis, the pre-understanding has been discussed continuously throughout the study.

Results were broadly consistent, and differences were discussed between the authors at investigators meetings, which thereby increases the study’s confirmability. Pre-understanding of a phenomenon includes both the theoretical knowledge and previous experiences but also the authors’ preconceived notions. Since the authors have been in contact with mobile care and cared for patients who have since been linked to mobile care, perceptions about mobile care have been created.

One limitation of the current study is that this is a relatively small study, and performed in a Swedish setting, which may affect transferability. However, this type of care is common in other countries such as Australia and England, for example, which makes our results transferable to those countries. although there can be some differences with regard to how mobile care is arranged.

Another limitation of the study was the number of participants. When the study started, we had planned to interview about 15 experts in each field. However, as some of them declined, the study ended with 12 completed interviews. Almost the same information was repeated within each expert group, which is why we experienced data saturation already after 9–10 interviews, i.e. a repeatable data within the same cohort of participants. For this reason, we believe that we have been able to capture the purpose of the study and thereby a good dependability.

Since the Covid-19 pandemic, healthcare has been forced to adopt new and innovative ways of working. We do not believe that the study’s findings would be outdated. On the contrary, this type of care is in line with modern healthcare, i.e. providing care at home instead of in hospitals. This provides a significant opportunity to reduce hospital-related visits and, therefore, a decrease in Covid-19-related infections.

## Conclusion

This study suggests that mobile care is seen as a health care that arrivesto the care seeker and not the opposite way. The resources will then be distributed where they do the most good, i.e. closest to the individual. Mobile care is also seen as a complement to traditional hospital care. This requires close collaboration between different staff categories and organizations, where there is no talk of boundaries but of the patient’s needs and situation instead. Mobile care is a way of care where it is necessary to work together to get a better holistic view where the care for the patient should be located Further research is needed to define or explain the concept of “mobile care”. In addition, more research is needed that examines how mobile care should be conducted. This can be done through interviews or surveys exploring the experiences of health care staff delivering mobile care and patients receiving it. Furthermore, there is also a need to study the effects and cost-effectiveness of mobile care compared to traditional emergency care.

## Data Availability

The datasets used and/or analysed during the current study are available from the corresponding author on reasonable request.
